# *trans*-Di-μ-acetato-κ^4^ *O*:*O*′-bis­[2-(5-phenyl­isoxazolin-3-yl)phenyl-κ^2^ *C* ^1^,*N*]dipalladium(II)

**DOI:** 10.1107/S1600536808014141

**Published:** 2008-05-17

**Authors:** Jin Zhou, Qibao Wang, Hongjian Sun

**Affiliations:** a School of Chemistry and Chemical Engineering, Shandong University, Shanda Nanlu 27, Jinan 250100, People’s Republic of China

## Abstract

The title compound, [Pd_2_(C_15_H_10_NO)_2_(C_2_H_3_O_2_)_2_], crystallized from a dichloro­methane/*n*-hexane solution with two crystallographically independent dimeric mol­ecules in the asymmetric unit. Each mol­ecule may be described as a dimer with an *anti* configuration and the cyclo­metallated fragments in the characteristic open-book disposition, linked by two bridging acetate ligands.

## Related literature

For a related palladacycle bridged by acetate ligands, see: Schultz *et al.* (2004 ▶). For related literature, see: Dupont *et al.* (2005 ▶).
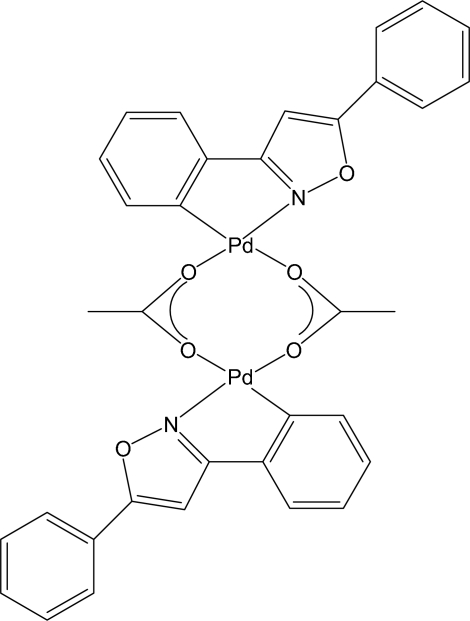


## Experimental

### 

#### Crystal data

[Pd_2_(C_15_H_10_NO)_2_(C_2_H_3_O_2_)_2_]
*M* *_r_* = 771.37
Monoclinic, 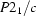

*a* = 14.8160 (6) Å
*b* = 24.2339 (10) Å
*c* = 19.6397 (8) Å
β = 103.233 (1)°
*V* = 6864.4 (5) Å^3^
*Z* = 8
Mo *K*α radiation
μ = 1.09 mm^−1^
*T* = 298 (2) K
0.28 × 0.20 × 0.15 mm

#### Data collection

Bruker SMART APEXII diffractometer
Absorption correction: multi-scan (*SADABS*; Sheldrick, 1996 ▶) *T*_min_ = 0.750, *T*_max_ = 0.854
80599 measured reflections
12098 independent reflections
8103 reflections with *I* > 2σ(*I*)
*R*_int_ = 0.038

#### Refinement

*R*[*F* ^2^ > 2σ(*F* ^2^)] = 0.049
*wR*(*F* ^2^) = 0.143
*S* = 1.09
12098 reflections
793 parameters
H-atom parameters constrained
Δρ_max_ = 0.81 e Å^−3^
Δρ_min_ = −0.58 e Å^−3^

### 

Data collection: *APEX2* (Bruker, 2005 ▶); cell refinement: *SAINT* (Bruker, 1997 ▶); data reduction: *SAINT*; program(s) used to solve structure: *SHELXS97* (Sheldrick, 2008 ▶); program(s) used to refine structure: *SHELXL97* (Sheldrick, 2008 ▶) and *PLATON* (Spek, 2003 ▶); molecular graphics: *SHELXTL* (Sheldrick, 2008 ▶); software used to prepare material for publication: *SHELXTL*.

## Supplementary Material

Crystal structure: contains datablocks I, global. DOI: 10.1107/S1600536808014141/cf2190sup1.cif

Structure factors: contains datablocks I. DOI: 10.1107/S1600536808014141/cf2190Isup2.hkl

Additional supplementary materials:  crystallographic information; 3D view; checkCIF report

## Figures and Tables

**Fig. 1.**
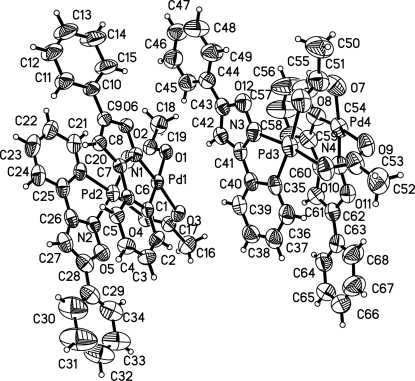
Molecular structure of the title compound. Displacement ellipsoids are drawn at 30% probability level.

## supplementary crystallographic information

### Comment

Recently, much attention is being paid to the use of palladacycles as catalyst precursors (Dupont *et al.*, 2005). Palladacycles bridged by acetate ligands have been reported (Schultz *et al.* 2004). We have prepared a new palladacycle by a C—H bond activation reaction of 3,5-diphenylisoxazole with Pd(OAc)_2_ in acetic acid. The asymmetric unit contains two molecules of the palladacycle having similar structures, of which only one will be discussed here. The molecular configuration of the complex is a dimeric form of the *anti* isomer with the cyclopalladated units in an arrangement linked by two acetate bridging ligands between the palladium atoms. The palladium atoms are coordinated in a slightly distorted square-planar arrangement by one cyclometallated isoxazole ligand and two bridging acetate ligands. In the crystal structure, the two cyclopalladated isoxazole subunits of the dimers are arranged in an *anti*-fashion. The chelating C, N-bonded isoxazoles are forced to lie above one another in the dimeric molecules because Pd1 and Pd2 are bridged by two *cis*-µ-acetate ligands. The resulting Pd—C (average 1.99 Å) and Pd—N (average 1.98 Å) bond distances fall within the range observed in other palladacycles (Smoliakova *et al.*, 2004). The Pd1···Pd2 distance [2.833 (5) Å] is noticeably larger than the sum of the covalent radii for two square-planar palladium(II) atoms, thus precluding any direct Pd—Pd interaction. The bond lengths of C7—N1 [1.303 (6) Å] and N2—C26 [1.318 (8) Å] are characteristic for C═N double bonds. The Pd—O bond distances *trans* to the carbon donor are slightly longer (*ca* 0.13 Å) than those *trans* to the nitrogen donor, as expected, because of the *trans* lengthening influence of carbon σ-donors.

### Experimental

3,5-Diphenylisoxazole (232 mg, 1.05 mmol) and Pd(OAc)_2_ (224 mg, 1.0 mmol) were added to 5 ml of acetic acid. The reaction mixture was stirred for 2 h at refluxing temperature. After removing all the volatiles, the orange residue was washed with diethyl ether, and was chromatographed on a silica gel column, eluting with CH_2_Cl_2_/*n*-hexane. The title compound was dried under vacuum, with a yield of 48% (185 mg). Crystals suitable for X-ray diffraction were obtained from the CH_2_Cl_2_/*n*-hexane solution.

### Refinement

All H atoms were positioned geometrically and treated as riding on their parent atoms with C—H = 0.93 Å (aromatic), 0.96 (methyl), 0.98 Å (methine), and with *U*_iso_(H) = 1.2 (1.5 for methyl groups) times *U*_eq_(C). Highly disordered and unidentified solvent molecules were treated with the SQUEEZE procedure of PLATON (Spek, 2003), as it proved impossible to model them with discrete atoms.

### Crystal data

**Table:**

[Pd_2_(C_15_H_10_NO)_2_(C_2_H_3_O_2_)_2_]	*F*_000_ = 3072
*M**_r_* = 771.37	*D*_x_ = 1.493 Mg m^−^^3^
Monoclinic, *P*2_1_/*c*	Mo *K*α radiation λ = 0.71073 Å
*a* = 14.8160 (6) Å	Cell parameters from 6715 reflections
*b* = 24.2339 (10) Å	θ = 2.2–24.8º
*c* = 19.6397 (8) Å	µ = 1.09 mm^−^^1^
β = 103.233 (1)º	*T* = 298 (2) K
*V* = 6864.4 (5) Å^3^	Block, yellow
*Z* = 8	0.28 × 0.20 × 0.15 mm

### Data collection

**Table:**

Bruker SMART APEXII diffractometer	12098 independent reflections
Radiation source: fine-focus sealed tube	8103 reflections with *I* > 2σ(*I*)
Monochromator: graphite	*R*_int_ = 0.039
*T* = 298(2) K	θ_max_ = 25.0º
φ and ω scans	θ_min_ = 1.4º
Absorption correction: multi-scan(SADABS; Sheldrick, 1996)	*h* = −17→17
*T*_min_ = 0.750, *T*_max_ = 0.854	*k* = 0→28
80599 measured reflections	*l* = 0→23

### Refinement

**Table:**

Refinement on *F*^2^	Secondary atom site location: difference Fourier map
Least-squares matrix: full	Hydrogen site location: inferred from neighbouring sites
*R*[*F*^2^ > 2σ(*F*^2^)] = 0.049	H-atom parameters constrained
*wR*(*F*^2^) = 0.143	*w* = 1/[σ^2^(*F*_o_^2^) + (0.0623*P*)^2^ + 7.5732*P*] where *P* = (*F*_o_^2^ + 2*F*_c_^2^)/3
*S* = 1.09	(Δ/σ)_max_ = 0.001
12098 reflections	Δρ_max_ = 0.81 e Å^−^^3^
793 parameters	Δρ_min_ = −0.58 e Å^−^^3^
Primary atom site location: structure-invariant direct methods	Extinction correction: none

### Special details

**Table:**

Geometry. All e.s.d.'s (except the e.s.d. in the dihedral angle between two l.s. planes) are estimated using the full covariance matrix. The cell e.s.d.'s are taken into account individually in the estimation of e.s.d.'s in distances, angles and torsion angles; correlations between e.s.d.'s in cell parameters are only used when they are defined by crystal symmetry. An approximate (isotropic) treatment of cell e.s.d.'s is used for estimating e.s.d.'s involving l.s. planes.
Refinement. Refinement of *F*^2^ against ALL reflections. The weighted *R*-factor *wR* and goodness of fit *S* are based on *F*^2^, conventional *R*-factors *R* are based on *F*, with *F* set to zero for negative *F*^2^. The threshold expression of *F*^2^ > σ(*F*^2^) is used only for calculating *R*-factors(gt) *etc*. and is not relevant to the choice of reflections for refinement. *R*-factors based on *F*^2^ are statistically about twice as large as those based on *F*, and *R*- factors based on ALL data will be even larger.

### Fractional atomic coordinates and isotropic or equivalent isotropic displacement parameters (Å^2^)

**Table:**

	*x*	*y*	*z*	*U*_iso_*/*U*_eq_	
Pd1	0.25845 (3)	0.545234 (17)	0.51711 (2)	0.06594 (13)	
Pd2	0.22368 (3)	0.62048 (2)	0.61849 (2)	0.08059 (16)	
Pd3	0.27410 (4)	0.36805 (3)	0.24384 (3)	0.1003 (2)	
Pd4	0.11212 (4)	0.35074 (3)	0.13585 (3)	0.1050 (2)	
C41	0.2228 (4)	0.4531 (3)	0.3289 (3)	0.0803 (16)	
O6	0.3757 (2)	0.63129 (15)	0.46051 (19)	0.0714 (9)	
C36	0.2313 (5)	0.3043 (4)	0.3647 (5)	0.107 (2)	
H36A	0.2458	0.2719	0.3442	0.129*	
C7	0.4499 (3)	0.5702 (2)	0.5345 (2)	0.0617 (12)	
O1	0.1543 (2)	0.59872 (17)	0.45465 (19)	0.0765 (10)	
O4	0.1169 (3)	0.5612 (2)	0.6166 (2)	0.0958 (13)	
C1	0.3602 (3)	0.4992 (2)	0.5727 (3)	0.0652 (12)	
O3	0.1587 (2)	0.49888 (17)	0.5463 (2)	0.0858 (11)	
N1	0.3682 (3)	0.58268 (18)	0.4966 (2)	0.0660 (10)	
C8	0.5150 (3)	0.6097 (2)	0.5236 (3)	0.0709 (14)	
H8A	0.5782	0.6104	0.5436	0.085*	
N2	0.3127 (3)	0.5885 (2)	0.6997 (3)	0.0857 (14)	
C9	0.4663 (3)	0.6461 (2)	0.4782 (3)	0.0700 (14)	
O5	0.3106 (3)	0.5401 (2)	0.7351 (2)	0.0970 (13)	
C17	0.1105 (4)	0.5155 (3)	0.5869 (3)	0.0850 (17)	
O11	0.0447 (3)	0.2742 (2)	0.2375 (3)	0.0999 (13)	
O12	0.2618 (3)	0.4951 (2)	0.2398 (2)	0.1017 (13)	
C19	0.1241 (4)	0.6422 (3)	0.4752 (4)	0.0794 (16)	
C6	0.4493 (3)	0.5203 (2)	0.5754 (2)	0.0647 (13)	
C35	0.2383 (4)	0.3549 (3)	0.3329 (3)	0.0902 (19)	
C4	0.5163 (4)	0.4469 (3)	0.6519 (3)	0.0861 (17)	
H4A	0.5679	0.4296	0.6795	0.103*	
N3	0.2566 (4)	0.4452 (3)	0.2733 (3)	0.0996 (16)	
O2	0.1436 (3)	0.66146 (18)	0.5361 (3)	0.0959 (12)	
N4	0.0365 (4)	0.3228 (3)	0.2003 (3)	0.0971 (16)	
O8	0.3198 (4)	0.3906 (3)	0.1518 (4)	0.1283 (19)	
C2	0.3520 (4)	0.4515 (2)	0.6092 (3)	0.0804 (16)	
H2A	0.2937	0.4368	0.6080	0.096*	
C26	0.3982 (5)	0.6078 (3)	0.7161 (3)	0.0907 (19)	
C60	−0.0044 (4)	0.3596 (3)	0.2330 (4)	0.0914 (18)	
C63	0.0116 (5)	0.2378 (3)	0.3431 (4)	0.108 (2)	
C27	0.4539 (5)	0.5722 (4)	0.7614 (4)	0.104 (2)	
H27A	0.5169	0.5760	0.7812	0.125*	
O9	0.1824 (4)	0.2750 (2)	0.1298 (3)	0.1223 (17)	
C40	0.2119 (4)	0.4029 (3)	0.3649 (3)	0.0853 (17)	
C20	0.3298 (4)	0.6723 (3)	0.6255 (4)	0.0850 (17)	
O10	0.2898 (4)	0.2855 (2)	0.2288 (3)	0.1221 (17)	
C5	0.5273 (4)	0.4942 (3)	0.6153 (3)	0.0763 (15)	
H5A	0.5861	0.5085	0.6173	0.092*	
C42	0.2032 (4)	0.5095 (3)	0.3326 (3)	0.0872 (17)	
H42A	0.1776	0.5266	0.3662	0.105*	
C62	0.0057 (4)	0.2836 (3)	0.2939 (4)	0.0949 (19)	
C43	0.2283 (4)	0.5344 (3)	0.2785 (3)	0.0872 (17)	
C39	0.1835 (5)	0.4005 (3)	0.4261 (4)	0.104 (2)	
H39A	0.1675	0.4327	0.4464	0.124*	
C10	0.4897 (4)	0.6960 (3)	0.4450 (3)	0.0874 (17)	
C3	0.4301 (4)	0.4254 (3)	0.6475 (3)	0.0889 (17)	
H3A	0.4238	0.3927	0.6707	0.107*	
C61	−0.0257 (4)	0.3358 (3)	0.2921 (4)	0.0950 (19)	
H61A	−0.0554	0.3525	0.3236	0.114*	
C25	0.4086 (4)	0.6570 (3)	0.6758 (4)	0.094 (2)	
C59	−0.0077 (5)	0.4129 (4)	0.2014 (4)	0.108 (2)	
C44	0.2302 (4)	0.5915 (3)	0.2552 (4)	0.0955 (19)	
C38	0.1784 (6)	0.3490 (4)	0.4581 (4)	0.116 (2)	
H38A	0.1589	0.3467	0.4998	0.139*	
C21	0.3336 (5)	0.7170 (3)	0.5853 (4)	0.103 (2)	
H21A	0.2815	0.7272	0.5515	0.123*	
C28	0.3978 (6)	0.5303 (4)	0.7713 (4)	0.104 (2)	
C45	0.1993 (5)	0.6326 (4)	0.2909 (4)	0.111 (2)	
H45A	0.1754	0.6241	0.3294	0.134*	
C24	0.4899 (5)	0.6874 (4)	0.6857 (4)	0.113 (2)	
H24A	0.5424	0.6773	0.7193	0.136*	
C11	0.5732 (5)	0.7217 (3)	0.4697 (4)	0.117 (3)	
H11A	0.6149	0.7070	0.5082	0.140*	
C54	0.0426 (6)	0.4168 (4)	0.1491 (4)	0.115 (2)	
C23	0.4898 (6)	0.7333 (4)	0.6437 (6)	0.133 (3)	
H23A	0.5432	0.7547	0.6501	0.159*	
C18	0.0554 (5)	0.6765 (3)	0.4213 (4)	0.117 (2)	
H18A	0.0447	0.6587	0.3765	0.175*	
H18B	0.0807	0.7126	0.4180	0.175*	
H18C	−0.0021	0.6797	0.4356	0.175*	
C15	0.4319 (6)	0.7169 (4)	0.3868 (5)	0.162 (4)	
H15A	0.3770	0.6988	0.3667	0.194*	
C16	0.0389 (5)	0.4753 (4)	0.5994 (5)	0.128 (3)	
H16A	0.0050	0.4914	0.6306	0.192*	
H16B	0.0690	0.4422	0.6198	0.192*	
H16C	−0.0030	0.4666	0.5557	0.192*	
C65	−0.0182 (9)	0.2019 (5)	0.4486 (5)	0.151 (4)	
H65A	−0.0459	0.2056	0.4864	0.181*	
C22	0.4148 (6)	0.7481 (3)	0.5937 (5)	0.120 (3)	
H22A	0.4172	0.7786	0.5654	0.144*	
C64	−0.0287 (6)	0.2431 (4)	0.3992 (5)	0.125 (3)	
H64A	−0.0630	0.2744	0.4037	0.150*	
C37	0.2023 (5)	0.3024 (4)	0.4275 (5)	0.120 (3)	
H37A	0.1993	0.2684	0.4491	0.143*	
C14	0.4553 (7)	0.7657 (5)	0.3574 (6)	0.201 (6)	
H14A	0.4147	0.7809	0.3187	0.241*	
C46	0.2028 (7)	0.6863 (4)	0.2709 (6)	0.136 (3)	
H46A	0.1805	0.7138	0.2957	0.163*	
C49	0.2652 (7)	0.6058 (5)	0.1991 (5)	0.147 (3)	
H49A	0.2861	0.5784	0.1733	0.176*	
C48	0.2699 (9)	0.6601 (6)	0.1805 (7)	0.179 (5)	
H48A	0.2956	0.6693	0.1430	0.215*	
C58	−0.0549 (7)	0.4573 (4)	0.2205 (6)	0.149 (4)	
H58A	−0.0873	0.4536	0.2556	0.179*	
C47	0.2378 (8)	0.7000 (5)	0.2160 (6)	0.141 (3)	
H47A	0.2399	0.7367	0.2027	0.169*	
C68	0.0618 (8)	0.1913 (4)	0.3356 (6)	0.154 (4)	
H68A	0.0869	0.1866	0.2967	0.185*	
C55	0.0464 (8)	0.4667 (5)	0.1173 (6)	0.171 (4)	
H55A	0.0805	0.4707	0.0832	0.206*	
C67	0.0736 (10)	0.1514 (5)	0.3883 (8)	0.177 (5)	
H67A	0.1109	0.1209	0.3859	0.212*	
C66	0.0319 (11)	0.1563 (5)	0.4431 (7)	0.176 (5)	
H66A	0.0379	0.1284	0.4763	0.211*	
C12	0.5961 (6)	0.7683 (4)	0.4390 (5)	0.143 (4)	
H12A	0.6538	0.7844	0.4560	0.172*	
C13	0.5374 (7)	0.7909 (5)	0.3852 (6)	0.165 (4)	
H13A	0.5523	0.8240	0.3665	0.198*	
C29	0.4113 (7)	0.4800 (4)	0.8146 (4)	0.117 (3)	
C34	0.3410 (9)	0.4440 (5)	0.8164 (6)	0.156 (4)	
H34A	0.2819	0.4514	0.7896	0.188*	
C33	0.3558 (12)	0.3960 (6)	0.8580 (7)	0.189 (5)	
H33A	0.3075	0.3718	0.8591	0.227*	
C30	0.4987 (9)	0.4668 (6)	0.8520 (7)	0.206 (6)	
H30A	0.5489	0.4893	0.8504	0.247*	
C31	0.5108 (13)	0.4185 (10)	0.8929 (11)	0.277 (12)	
H31A	0.5696	0.4091	0.9187	0.332*	
C57	−0.0535 (11)	0.5061 (6)	0.1877 (7)	0.228 (8)	
H57A	−0.0869	0.5359	0.1990	0.274*	
C56	−0.0020 (12)	0.5119 (5)	0.1368 (8)	0.247 (8)	
H56A	0.0005	0.5460	0.1156	0.296*	
O7	0.1818 (5)	0.3897 (3)	0.0727 (3)	0.138 (2)	
C51	0.2666 (8)	0.3996 (4)	0.0921 (6)	0.127 (3)	
C53	0.2457 (7)	0.2581 (4)	0.1773 (5)	0.122 (3)	
C52	0.2749 (8)	0.1985 (4)	0.1740 (6)	0.174 (4)	
H52A	0.2375	0.1813	0.1331	0.262*	
H52B	0.2669	0.1794	0.2150	0.262*	
H52C	0.3389	0.1970	0.1718	0.262*	
C50	0.3113 (9)	0.4249 (5)	0.0377 (6)	0.196 (5)	
H50A	0.2651	0.4309	−0.0047	0.294*	
H50B	0.3581	0.4004	0.0286	0.294*	
H50C	0.3392	0.4595	0.0546	0.294*	
C32	0.4400 (14)	0.3869 (7)	0.8950 (8)	0.192 (7)	
H32A	0.4500	0.3562	0.9242	0.230*	

### Atomic displacement parameters (Å^2^)

**Table:**

	*U*^11^	*U*^22^	*U*^33^	*U*^12^	*U*^13^	*U*^23^
Pd1	0.0508 (2)	0.0751 (3)	0.0743 (3)	−0.00960 (18)	0.01948 (17)	−0.0122 (2)
Pd2	0.0638 (3)	0.0948 (3)	0.0854 (3)	−0.0040 (2)	0.0218 (2)	−0.0239 (2)
Pd3	0.0736 (3)	0.1213 (4)	0.1066 (4)	0.0092 (3)	0.0214 (3)	−0.0357 (3)
Pd4	0.0874 (4)	0.1304 (5)	0.0962 (4)	0.0143 (3)	0.0189 (3)	−0.0161 (3)
C41	0.059 (3)	0.101 (5)	0.084 (4)	−0.008 (3)	0.023 (3)	−0.032 (4)
O6	0.052 (2)	0.087 (3)	0.077 (2)	−0.0084 (17)	0.0183 (16)	0.0039 (19)
C36	0.078 (4)	0.114 (6)	0.110 (6)	0.004 (4)	−0.021 (4)	−0.013 (5)
C7	0.056 (3)	0.074 (3)	0.059 (3)	0.002 (2)	0.021 (2)	−0.008 (2)
O1	0.055 (2)	0.091 (3)	0.080 (2)	−0.0064 (19)	0.0069 (17)	−0.007 (2)
O4	0.063 (2)	0.129 (4)	0.103 (3)	−0.019 (2)	0.036 (2)	−0.012 (3)
C1	0.061 (3)	0.070 (3)	0.065 (3)	−0.007 (2)	0.016 (2)	−0.018 (3)
O3	0.061 (2)	0.092 (3)	0.108 (3)	−0.0222 (19)	0.027 (2)	−0.009 (2)
N1	0.055 (2)	0.074 (3)	0.074 (3)	−0.0017 (19)	0.024 (2)	−0.003 (2)
C8	0.049 (3)	0.091 (4)	0.074 (3)	−0.007 (3)	0.016 (2)	−0.002 (3)
N2	0.079 (3)	0.101 (4)	0.078 (3)	−0.003 (3)	0.019 (3)	−0.016 (3)
C9	0.054 (3)	0.091 (4)	0.069 (3)	−0.011 (3)	0.022 (2)	−0.004 (3)
O5	0.092 (3)	0.119 (4)	0.083 (3)	0.007 (3)	0.026 (2)	−0.011 (3)
C17	0.050 (3)	0.116 (5)	0.091 (4)	−0.024 (3)	0.019 (3)	0.000 (4)
O11	0.091 (3)	0.099 (3)	0.104 (3)	0.003 (2)	0.011 (3)	−0.016 (3)
O12	0.092 (3)	0.118 (4)	0.103 (3)	−0.009 (3)	0.039 (3)	−0.024 (3)
C19	0.050 (3)	0.091 (4)	0.095 (4)	−0.005 (3)	0.014 (3)	−0.003 (4)
C6	0.064 (3)	0.080 (3)	0.054 (3)	−0.001 (3)	0.021 (2)	−0.018 (3)
C35	0.061 (3)	0.109 (5)	0.089 (4)	0.002 (3)	−0.007 (3)	−0.025 (4)
C4	0.077 (4)	0.092 (4)	0.090 (4)	0.016 (3)	0.021 (3)	0.008 (3)
N3	0.085 (4)	0.106 (4)	0.111 (4)	−0.009 (3)	0.029 (3)	−0.027 (4)
O2	0.078 (3)	0.097 (3)	0.108 (3)	0.006 (2)	0.012 (2)	−0.020 (3)
N4	0.079 (3)	0.105 (4)	0.103 (4)	0.011 (3)	0.012 (3)	−0.011 (4)
O8	0.102 (4)	0.159 (5)	0.138 (5)	0.007 (3)	0.058 (4)	−0.041 (4)
C2	0.077 (4)	0.068 (4)	0.105 (4)	−0.009 (3)	0.040 (3)	−0.008 (3)
C26	0.077 (4)	0.118 (6)	0.076 (4)	0.006 (4)	0.016 (3)	−0.034 (4)
C60	0.070 (4)	0.106 (5)	0.090 (5)	0.013 (3)	0.001 (3)	−0.012 (4)
C63	0.107 (5)	0.097 (6)	0.114 (6)	−0.013 (4)	0.016 (4)	−0.018 (5)
C27	0.086 (5)	0.137 (7)	0.090 (5)	0.011 (5)	0.018 (4)	−0.032 (5)
O9	0.115 (4)	0.133 (4)	0.118 (4)	0.025 (3)	0.024 (3)	−0.041 (3)
C40	0.062 (3)	0.111 (5)	0.079 (4)	−0.006 (3)	0.007 (3)	−0.024 (4)
C20	0.080 (4)	0.084 (4)	0.094 (4)	−0.006 (3)	0.026 (3)	−0.033 (4)
O10	0.110 (4)	0.123 (4)	0.124 (4)	0.033 (3)	0.006 (3)	−0.038 (3)
C5	0.060 (3)	0.095 (4)	0.075 (3)	−0.001 (3)	0.017 (3)	−0.008 (3)
C42	0.068 (3)	0.112 (5)	0.081 (4)	−0.003 (3)	0.017 (3)	−0.018 (4)
C62	0.073 (4)	0.110 (6)	0.097 (5)	0.001 (4)	0.010 (3)	−0.014 (4)
C43	0.064 (3)	0.111 (5)	0.084 (4)	−0.003 (3)	0.010 (3)	−0.015 (4)
C39	0.094 (5)	0.115 (6)	0.097 (5)	−0.005 (4)	0.012 (4)	−0.030 (4)
C10	0.071 (4)	0.104 (5)	0.090 (4)	−0.015 (3)	0.027 (3)	0.016 (4)
C3	0.087 (4)	0.084 (4)	0.100 (4)	0.009 (3)	0.030 (4)	0.014 (3)
C61	0.073 (4)	0.116 (6)	0.096 (5)	0.017 (4)	0.019 (3)	−0.004 (4)
C25	0.072 (4)	0.113 (5)	0.100 (5)	−0.007 (4)	0.025 (4)	−0.043 (4)
C59	0.090 (5)	0.125 (6)	0.109 (5)	0.029 (4)	0.023 (4)	−0.003 (5)
C44	0.073 (4)	0.117 (6)	0.096 (5)	−0.004 (4)	0.017 (3)	0.001 (4)
C38	0.109 (6)	0.135 (7)	0.097 (5)	−0.009 (5)	0.008 (4)	0.001 (5)
C21	0.087 (5)	0.105 (5)	0.116 (5)	−0.006 (4)	0.021 (4)	−0.028 (5)
C28	0.112 (6)	0.120 (6)	0.084 (5)	0.033 (5)	0.027 (4)	−0.018 (4)
C45	0.113 (6)	0.111 (6)	0.113 (6)	0.001 (5)	0.031 (5)	0.002 (5)
C24	0.085 (5)	0.137 (7)	0.116 (6)	−0.021 (5)	0.020 (4)	−0.043 (5)
C11	0.074 (4)	0.141 (6)	0.131 (6)	−0.029 (4)	0.014 (4)	0.033 (5)
C54	0.106 (5)	0.125 (7)	0.115 (6)	0.027 (5)	0.026 (5)	0.009 (5)
C23	0.103 (6)	0.143 (8)	0.160 (8)	−0.045 (6)	0.045 (6)	−0.052 (7)
C18	0.092 (5)	0.118 (6)	0.131 (6)	0.014 (4)	0.008 (4)	0.003 (5)
C15	0.112 (6)	0.201 (10)	0.156 (8)	−0.061 (6)	−0.004 (6)	0.094 (8)
C16	0.079 (4)	0.164 (7)	0.148 (7)	−0.048 (5)	0.039 (4)	0.003 (6)
C65	0.202 (11)	0.145 (9)	0.107 (7)	−0.016 (8)	0.038 (7)	0.003 (7)
C22	0.124 (7)	0.102 (5)	0.137 (7)	−0.030 (5)	0.036 (6)	−0.025 (5)
C64	0.145 (7)	0.119 (7)	0.108 (6)	0.005 (5)	0.022 (5)	−0.015 (5)
C37	0.098 (5)	0.126 (7)	0.114 (6)	0.004 (5)	−0.018 (5)	−0.007 (5)
C14	0.116 (7)	0.268 (14)	0.194 (10)	−0.071 (8)	−0.013 (7)	0.138 (10)
C46	0.138 (8)	0.117 (7)	0.144 (8)	0.010 (6)	0.016 (6)	0.014 (6)
C49	0.187 (10)	0.140 (8)	0.135 (7)	0.009 (7)	0.080 (7)	0.009 (6)
C48	0.218 (13)	0.181 (12)	0.157 (10)	0.007 (10)	0.082 (9)	0.050 (9)
C58	0.167 (9)	0.130 (7)	0.172 (9)	0.056 (6)	0.084 (7)	0.032 (7)
C47	0.148 (8)	0.134 (8)	0.131 (8)	−0.005 (7)	0.012 (7)	0.038 (7)
C68	0.206 (11)	0.101 (6)	0.169 (9)	0.017 (7)	0.073 (8)	0.005 (7)
C55	0.192 (11)	0.159 (9)	0.194 (11)	0.063 (8)	0.107 (9)	0.041 (8)
C67	0.242 (14)	0.119 (8)	0.185 (12)	0.017 (8)	0.081 (11)	0.010 (9)
C66	0.259 (16)	0.112 (8)	0.140 (9)	0.007 (9)	0.010 (10)	0.010 (7)
C12	0.087 (5)	0.173 (9)	0.167 (8)	−0.046 (5)	0.025 (5)	0.056 (7)
C13	0.122 (7)	0.187 (10)	0.189 (10)	−0.048 (7)	0.040 (7)	0.080 (8)
C29	0.118 (6)	0.150 (8)	0.090 (5)	0.034 (6)	0.040 (5)	−0.011 (5)
C34	0.163 (10)	0.178 (11)	0.130 (8)	0.039 (9)	0.039 (7)	0.017 (8)
C33	0.232 (16)	0.186 (12)	0.172 (11)	0.064 (11)	0.091 (11)	0.058 (10)
C30	0.144 (10)	0.238 (14)	0.226 (13)	0.075 (10)	0.024 (9)	0.071 (12)
C31	0.175 (15)	0.35 (3)	0.31 (2)	0.122 (17)	0.063 (15)	0.17 (2)
C57	0.325 (18)	0.177 (11)	0.244 (14)	0.130 (12)	0.192 (14)	0.082 (11)
C56	0.339 (19)	0.170 (11)	0.296 (17)	0.126 (13)	0.206 (17)	0.117 (12)
O7	0.114 (4)	0.193 (6)	0.114 (4)	0.019 (4)	0.040 (4)	−0.004 (4)
C51	0.134 (8)	0.141 (7)	0.124 (7)	0.021 (6)	0.065 (7)	−0.018 (6)
C53	0.118 (6)	0.135 (7)	0.116 (6)	0.031 (6)	0.030 (5)	−0.032 (6)
C52	0.214 (11)	0.130 (8)	0.180 (10)	0.058 (8)	0.046 (9)	−0.035 (7)
C50	0.227 (13)	0.232 (13)	0.165 (9)	0.017 (10)	0.120 (10)	0.014 (9)
C32	0.243 (18)	0.196 (14)	0.157 (10)	0.124 (14)	0.087 (13)	0.055 (10)

### Geometric parameters (Å, °)

**Table:**

Pd1—N1	1.983 (4)	C61—H61A	0.930
Pd1—C1	1.989 (5)	C25—C24	1.386 (10)
Pd1—O3	2.041 (4)	C59—C58	1.382 (11)
Pd1—O1	2.168 (4)	C59—C54	1.404 (10)
Pd2—N2	1.980 (5)	C44—C45	1.355 (10)
Pd2—C20	1.992 (6)	C44—C49	1.365 (11)
Pd2—O2	2.033 (5)	C38—C37	1.364 (11)
Pd2—O4	2.131 (4)	C38—H38A	0.930
Pd3—C35	1.966 (7)	C21—C22	1.397 (10)
Pd3—N3	1.991 (6)	C21—H21A	0.930
Pd3—O10	2.044 (5)	C28—C29	1.475 (12)
Pd3—O8	2.141 (6)	C45—C46	1.363 (11)
Pd4—C54	1.954 (8)	C45—H45A	0.930
Pd4—N4	1.990 (6)	C24—C23	1.385 (12)
Pd4—O7	2.021 (6)	C24—H24A	0.930
Pd4—O9	2.128 (5)	C11—C12	1.360 (10)
C41—N3	1.315 (8)	C11—H11A	0.930
C41—C42	1.404 (9)	C54—C55	1.368 (12)
C41—C40	1.434 (9)	C23—C22	1.352 (12)
O6—C9	1.355 (6)	C23—H23A	0.930
O6—N1	1.392 (5)	C18—H18A	0.960
C36—C35	1.391 (10)	C18—H18B	0.960
C36—C37	1.397 (11)	C18—H18C	0.960
C36—H36A	0.930	C15—C14	1.392 (12)
C7—N1	1.303 (6)	C15—H15A	0.930
C7—C8	1.408 (7)	C16—H16A	0.960
C7—C6	1.454 (7)	C16—H16B	0.960
O1—C19	1.247 (7)	C16—H16C	0.960
O4—C17	1.245 (8)	C65—C66	1.351 (15)
C1—C2	1.380 (8)	C65—C64	1.375 (12)
C1—C6	1.406 (7)	C65—H65A	0.930
O3—C17	1.251 (7)	C22—H22A	0.930
C8—C9	1.340 (7)	C64—H64A	0.930
C8—H8A	0.930	C37—H37A	0.930
N2—C26	1.318 (8)	C14—C13	1.360 (13)
N2—O5	1.368 (7)	C14—H14A	0.930
C9—C10	1.454 (8)	C46—C47	1.341 (13)
O5—C28	1.345 (8)	C46—H46A	0.930
C17—C16	1.502 (8)	C49—C48	1.371 (15)
O11—N4	1.376 (7)	C49—H49A	0.930
O11—C62	1.381 (8)	C48—C47	1.341 (14)
O12—C43	1.380 (7)	C48—H48A	0.930
O12—N3	1.389 (7)	C58—C57	1.348 (13)
C19—O2	1.254 (7)	C58—H58A	0.930
C19—C18	1.535 (9)	C47—H47A	0.930
C6—C5	1.390 (7)	C68—C67	1.397 (14)
C35—C40	1.418 (9)	C68—H68A	0.930
C4—C3	1.364 (8)	C55—C56	1.410 (14)
C4—C5	1.383 (8)	C55—H55A	0.930
C4—H4A	0.930	C67—C66	1.364 (16)
N4—C60	1.325 (8)	C67—H67A	0.930
O8—C51	1.273 (11)	C66—H66A	0.930
C2—C3	1.379 (8)	C12—C13	1.324 (12)
C2—H2A	0.930	C12—H12A	0.930
C26—C27	1.372 (10)	C13—H13A	0.930
C26—C25	1.460 (10)	C29—C34	1.363 (13)
C60—C61	1.396 (9)	C29—C30	1.372 (13)
C60—C59	1.428 (10)	C34—C33	1.409 (15)
C63—C64	1.373 (11)	C34—H34A	0.930
C63—C68	1.377 (11)	C33—C32	1.312 (18)
C63—C62	1.460 (11)	C33—H33A	0.930
C27—C28	1.354 (11)	C30—C31	1.41 (2)
C27—H27A	0.930	C30—H30A	0.930
O9—C53	1.231 (9)	C31—C32	1.31 (2)
C40—C39	1.363 (9)	C31—H31A	0.930
C20—C21	1.349 (10)	C57—C56	1.398 (15)
C20—C25	1.396 (9)	C57—H57A	0.930
O10—C53	1.260 (9)	C56—H56A	0.930
C5—H5A	0.930	O7—C51	1.250 (11)
C42—C43	1.347 (9)	C51—C50	1.511 (13)
C42—H42A	0.930	C53—C52	1.513 (12)
C62—C61	1.347 (9)	C52—H52A	0.960
C43—C44	1.460 (10)	C52—H52B	0.960
C39—C38	1.409 (11)	C52—H52C	0.960
C39—H39A	0.930	C50—H50A	0.960
C10—C15	1.360 (10)	C50—H50B	0.960
C10—C11	1.370 (9)	C50—H50C	0.960
C3—H3A	0.930	C32—H32A	0.930
			
N1—Pd1—C1	79.3 (2)	C45—C44—C43	120.1 (7)
N1—Pd1—O3	171.81 (17)	C49—C44—C43	122.0 (8)
C1—Pd1—O3	92.49 (19)	C37—C38—C39	119.5 (8)
N1—Pd1—O1	97.02 (16)	C37—C38—H38A	120.3
C1—Pd1—O1	176.35 (18)	C39—C38—H38A	120.3
O3—Pd1—O1	91.16 (15)	C20—C21—C22	121.0 (8)
N2—Pd2—C20	79.7 (3)	C20—C21—H21A	119.5
N2—Pd2—O2	172.7 (2)	C22—C21—H21A	119.5
C20—Pd2—O2	93.1 (3)	O5—C28—C27	109.8 (7)
N2—Pd2—O4	96.0 (2)	O5—C28—C29	116.0 (8)
C20—Pd2—O4	175.7 (3)	C27—C28—C29	134.1 (9)
O2—Pd2—O4	91.14 (18)	C44—C45—C46	120.8 (9)
C35—Pd3—N3	79.3 (3)	C44—C45—H45A	119.6
C35—Pd3—O10	91.8 (3)	C46—C45—H45A	119.6
N3—Pd3—O10	171.0 (3)	C23—C24—C25	117.4 (8)
C35—Pd3—O8	173.7 (3)	C23—C24—H24A	121.3
N3—Pd3—O8	95.3 (2)	C25—C24—H24A	121.3
O10—Pd3—O8	93.5 (2)	C12—C11—C10	121.1 (7)
C54—Pd4—N4	78.7 (3)	C12—C11—H11A	119.4
C54—Pd4—O7	93.3 (3)	C10—C11—H11A	119.4
N4—Pd4—O7	171.9 (3)	C55—C54—C59	118.7 (8)
C54—Pd4—O9	174.1 (3)	C55—C54—Pd4	125.8 (7)
N4—Pd4—O9	95.5 (2)	C59—C54—Pd4	115.2 (6)
O7—Pd4—O9	92.5 (2)	C22—C23—C24	122.3 (8)
N3—C41—C42	107.9 (6)	C22—C23—H23A	118.8
N3—C41—C40	113.1 (6)	C24—C23—H23A	118.8
C42—C41—C40	138.9 (6)	C19—C18—H18A	109.5
C9—O6—N1	106.4 (4)	C19—C18—H18B	109.5
C35—C36—C37	119.7 (8)	H18A—C18—H18B	109.5
C35—C36—H36A	120.1	C19—C18—H18C	109.5
C37—C36—H36A	120.1	H18A—C18—H18C	109.5
N1—C7—C8	109.5 (5)	H18B—C18—H18C	109.5
N1—C7—C6	112.7 (4)	C10—C15—C14	119.8 (8)
C8—C7—C6	137.8 (5)	C10—C15—H15A	120.1
C19—O1—Pd1	125.7 (4)	C14—C15—H15A	120.1
C17—O4—Pd2	125.8 (4)	C17—C16—H16A	109.5
C2—C1—C6	118.6 (5)	C17—C16—H16B	109.5
C2—C1—Pd1	127.6 (4)	H16A—C16—H16B	109.5
C6—C1—Pd1	113.8 (4)	C17—C16—H16C	109.5
C17—O3—Pd1	123.9 (4)	H16A—C16—H16C	109.5
C7—N1—O6	108.3 (4)	H16B—C16—H16C	109.5
C7—N1—Pd1	118.4 (4)	C66—C65—C64	120.8 (11)
O6—N1—Pd1	131.0 (3)	C66—C65—H65A	119.6
C9—C8—C7	105.4 (5)	C64—C65—H65A	119.6
C9—C8—H8A	127.3	C23—C22—C21	119.0 (9)
C7—C8—H8A	127.3	C23—C22—H22A	120.5
C26—N2—O5	108.1 (6)	C21—C22—H22A	120.5
C26—N2—Pd2	118.9 (5)	C63—C64—C65	120.0 (9)
O5—N2—Pd2	131.0 (4)	C63—C64—H64A	120.0
C8—C9—O6	110.3 (5)	C65—C64—H64A	120.0
C8—C9—C10	134.3 (5)	C38—C37—C36	121.7 (9)
O6—C9—C10	115.4 (5)	C38—C37—H37A	119.2
C28—O5—N2	106.8 (6)	C36—C37—H37A	119.2
O4—C17—O3	126.2 (5)	C13—C14—C15	120.0 (9)
O4—C17—C16	118.8 (6)	C13—C14—H14A	120.0
O3—C17—C16	115.0 (7)	C15—C14—H14A	120.0
N4—O11—C62	106.5 (5)	C47—C46—C45	121.0 (10)
C43—O12—N3	105.9 (5)	C47—C46—H46A	119.5
O1—C19—O2	127.0 (6)	C45—C46—H46A	119.5
O1—C19—C18	117.7 (6)	C44—C49—C48	120.6 (10)
O2—C19—C18	115.3 (6)	C44—C49—H49A	119.7
C5—C6—C1	120.6 (5)	C48—C49—H49A	119.7
C5—C6—C7	125.4 (5)	C47—C48—C49	120.6 (11)
C1—C6—C7	114.0 (5)	C47—C48—H48A	119.7
C36—C35—C40	117.8 (7)	C49—C48—H48A	119.7
C36—C35—Pd3	127.3 (6)	C57—C58—C59	119.3 (9)
C40—C35—Pd3	114.7 (6)	C57—C58—H58A	120.3
C3—C4—C5	120.2 (6)	C59—C58—H58A	120.3
C3—C4—H4A	119.9	C48—C47—C46	119.1 (11)
C5—C4—H4A	119.9	C48—C47—H47A	120.4
C41—N3—O12	109.9 (5)	C46—C47—H47A	120.4
C41—N3—Pd3	118.5 (5)	C63—C68—C67	117.8 (10)
O12—N3—Pd3	130.9 (4)	C63—C68—H68A	121.1
C19—O2—Pd2	122.8 (4)	C67—C68—H68A	121.1
C60—N4—O11	108.2 (6)	C54—C55—C56	119.0 (10)
C60—N4—Pd4	117.7 (5)	C54—C55—H55A	120.5
O11—N4—Pd4	129.4 (4)	C56—C55—H55A	120.5
C51—O8—Pd3	124.9 (6)	C66—C67—C68	121.5 (12)
C3—C2—C1	120.3 (5)	C66—C67—H67A	119.2
C3—C2—H2A	119.9	C68—C67—H67A	119.2
C1—C2—H2A	119.9	C65—C66—C67	119.4 (12)
N2—C26—C27	109.8 (7)	C65—C66—H66A	120.3
N2—C26—C25	111.8 (6)	C67—C66—H66A	120.3
C27—C26—C25	138.0 (7)	C13—C12—C11	120.9 (8)
N4—C60—C61	109.9 (7)	C13—C12—H12A	119.6
N4—C60—C59	111.9 (7)	C11—C12—H12A	119.6
C61—C60—C59	138.0 (7)	C12—C13—C14	119.8 (9)
C64—C63—C68	120.4 (9)	C12—C13—H13A	120.1
C64—C63—C62	119.3 (8)	C14—C13—H13A	120.1
C68—C63—C62	120.2 (9)	C34—C29—C30	118.0 (11)
C28—C27—C26	105.4 (7)	C34—C29—C28	122.7 (9)
C28—C27—H27A	127.3	C30—C29—C28	119.2 (11)
C26—C27—H27A	127.3	C29—C34—C33	121.6 (13)
C53—O9—Pd4	122.8 (5)	C29—C34—H34A	119.2
C39—C40—C35	121.9 (7)	C33—C34—H34A	119.2
C39—C40—C41	124.1 (7)	C32—C33—C34	117.5 (16)
C35—C40—C41	113.8 (6)	C32—C33—H33A	121.2
C21—C20—C25	119.1 (6)	C34—C33—H33A	121.2
C21—C20—Pd2	127.1 (5)	C29—C30—C31	118.8 (15)
C25—C20—Pd2	113.6 (6)	C29—C30—H30A	120.6
C53—O10—Pd3	125.2 (5)	C31—C30—H30A	120.6
C4—C5—C6	119.2 (5)	C32—C31—C30	120.4 (16)
C4—C5—H5A	120.4	C32—C31—H31A	119.8
C6—C5—H5A	120.4	C30—C31—H31A	119.8
C43—C42—C41	107.3 (6)	C58—C57—C56	120.0 (10)
C43—C42—H42A	126.4	C58—C57—H57A	120.0
C41—C42—H42A	126.4	C56—C57—H57A	120.0
C61—C62—O11	109.8 (7)	C57—C56—C55	120.8 (11)
C61—C62—C63	134.4 (7)	C57—C56—H56A	119.6
O11—C62—C63	115.7 (7)	C55—C56—H56A	119.6
C42—C43—O12	109.0 (6)	C51—O7—Pd4	121.4 (6)
C42—C43—C44	134.4 (7)	O7—C51—O8	128.1 (9)
O12—C43—C44	116.5 (6)	O7—C51—C50	115.8 (10)
C40—C39—C38	119.3 (7)	O8—C51—C50	116.2 (10)
C40—C39—H39A	120.4	O9—C53—O10	126.7 (8)
C38—C39—H39A	120.4	O9—C53—C52	117.4 (9)
C15—C10—C11	118.2 (6)	O10—C53—C52	115.8 (9)
C15—C10—C9	121.3 (6)	C53—C52—H52A	109.5
C11—C10—C9	120.5 (6)	C53—C52—H52B	109.5
C4—C3—C2	121.1 (6)	H52A—C52—H52B	109.5
C4—C3—H3A	119.5	C53—C52—H52C	109.5
C2—C3—H3A	119.5	H52A—C52—H52C	109.5
C62—C61—C60	105.6 (6)	H52B—C52—H52C	109.5
C62—C61—H61A	127.2	C51—C50—H50A	109.5
C60—C61—H61A	127.2	C51—C50—H50B	109.5
C24—C25—C20	121.2 (8)	H50A—C50—H50B	109.5
C24—C25—C26	123.7 (8)	C51—C50—H50C	109.5
C20—C25—C26	115.2 (6)	H50A—C50—H50C	109.5
C58—C59—C54	122.1 (9)	H50B—C50—H50C	109.5
C58—C59—C60	123.8 (8)	C31—C32—C33	123.5 (17)
C54—C59—C60	114.1 (7)	C31—C32—H32A	118.2
C45—C44—C49	117.8 (8)	C33—C32—H32A	118.2
